# Bibliometric analysis of microRNAs and Parkinson’s disease from 2014 to 2023

**DOI:** 10.3389/fneur.2024.1466186

**Published:** 2024-09-25

**Authors:** Lingshan Chen, Jianfei Chen, Wei Weng, Min Wu, Xueping Zhou, Pingkang Yan

**Affiliations:** ^1^Medical Laboratory Specialty, The Second Hospital of Jinhua, Jinhua, Zhejiang, China; ^2^School of Medical Technology and Information Engineering, Zhejiang Chinese Medical University, Hangzhou, China; ^3^Gerontology Department, The Second Hospital of Jinhua, Jinhua, Zhejiang, China

**Keywords:** microRNAs, Parkinson’s disease, bibliometric analysis, Bibliometrix R, VOSviewer

## Abstract

**Background:**

Parkinson’s disease (PD) is a neurodegenerative disorder characterized by the degeneration of dopaminergic neurons. Recent research has emphasized a significant correlation between microRNAs (miRNAs) and PD. To identify key research areas, provide a comprehensive overview of current research in various fields, and propose potential directions for future studies, a bibliometric analysis was conducted on the involvement of miRNAs in Parkinson’s disease from 2014 to 2023.

**Methods:**

Relevant literature records were collected from the Web of Science Core Collection on February 29, 2024. Subsequently, the data underwent analysis using the Bibliometrix R package and VOSviewer (version 1.6.19).

**Results:**

The annual scientific publications on miRNAs and Parkinson’s disease demonstrated an increasing trend, with an annual growth rate of 12.67%. China, the United States, and India emerged as the top three most productive countries/regions. The University of Barcelona had the highest annual publications, followed by Central South University and the Helmholtz Association. The *INTERNATIONAL JOURNAL OF MOLECULAR SCIENCES* held the top position in terms of H-index and total citations, reflecting its extensive influence and prolific publication output. *Kim, J., Junn, E., Hébert, S.S.,* and *Doxakis, E.* were the most frequently co-cited authors in the field. Based on the analysis of keywords, the most frequently occurring terms included “alpha-synuclein,” “neurodegenerative disease,” “exosome,” “neuroinflammation,” “oxidative stress,” “autophagy,” and “amyotrophic lateral sclerosis,” which have emerged as prominent research topics. Concurrently, there has been notable interest in topics such as “ceRNA,” “lncRNAs,” “mitochondrial dysfunction,” and “circular RNA.”

**Conclusion:**

This study focused on identifying emerging trends and critical research topics in the bibliometric analysis of microRNAs related to Parkinson’s disease. These findings highlight the diverse research landscape and evolving trend of miRNA-related research in PD. The field of miRNA research in Parkinson’s disease is actively exploring the underlying mechanisms of miRNA function, identifying potential diagnostic markers, and developing innovative therapeutic strategies. The results of our study offer significant contributions to researchers’ ability to track contemporary developments and guide the trajectory of future research in this domain.

## Introduction

Parkinson’s disease (PD) is a neurodegenerative disorder that is rapidly increasing in prevalence. It has become the second most common condition of this type, affecting over 6 million individuals worldwide. This number has grown by 2.5 times in the past 30 years alone, making Parkinson’s disease a leading cause of neurological disability (1). The incidence of Parkinson’s disease is rising more rapidly than any other neurological disorder, resulting in higher rates of disability and mortality (2).

PD is a multifactorial disease resulting from the complex interplay between genetic and epigenetic factors. Recent research has shed light on the epigenetic mechanisms that influence pathways related to the development of PD, including DNA methylation, histone post-translational modifications, and microRNA (miRNA or miR) regulation (3). MicroRNAs are small non-coding RNAs, typically 19–25 nucleotides in length (4). They function as post-transcriptional regulators of gene expression, playing crucial physiological and pathological roles (5). In recent years, miRNAs have garnered significant attention for their involvement in PD pathogenesis (6). These small non-coding RNAs exert a pivotal influence on post-transcriptional gene regulation and various cellular processes (4). It is now understood that miRNAs frequently target genes associated with PD risk, affecting key pathways involved in neuronal function and survival (7).

There has been a substantial increase in the exploration of miRNAs in the context of PD in recent years (4). To gain a comprehensive understanding of this field, bibliometric visualization analysis has emerged as a valuable tool, providing insights into the trends, patterns, and key contributors shaping this research landscape (8–10). Indeed, it is crucial for researchers, clinicians, and policymakers to comprehend the dynamics and advancements in the exploration of miRNAs in Parkinson’s disease through bibliometric visualization. By understanding global trends and collaborative networks, we can derive valuable insights that contribute meaningfully to the field. Herein, we conducted bibliometric visualization to gain a deeper understanding of miRNAs in PD.

## Materials and methods

### Bibliographical sources

The Web of Science Core Collection (WoSCC) provides a comprehensive platform for the assessment of scientific metrics in a wide array of academic disciplines (11, 12). Its principles include data structuring, subject categorization, citation analysis, an international perspective, and scalability. It is widely recognized as the leading database for bibliometric analysis (13). Previous studies have demonstrated the effectiveness of bibliometric analysis conducted using the WoSCC database (14, 15). The present study obtained relevant data from the Science Citation Index Expanded (SCI-EXPANDED) within the Web of Science Core Collection, spanning from January 1, 2014 to December 31, 2023. Limiting the search to a decade enhanced the efficiency and feasibility of data analysis and visualization, ultimately reducing the complexity and cost associated with data processing. Searches utilized the following MeSH terms: “Parkinson disease” and “microRNA” (as detailed in Supplementary material 1) were employed to search the title (TI), abstract (AB), and author keywords (AK) for more precise results (16, 17). As previously mentioned, we extracted and exported both articles and review articles in either “Plain text file” or “Tab-delimited file” formats. The data was documented as “Full record and cited references” to ensure comprehensive coverage (16, 18).

### Data analysis

**Figure 1:**
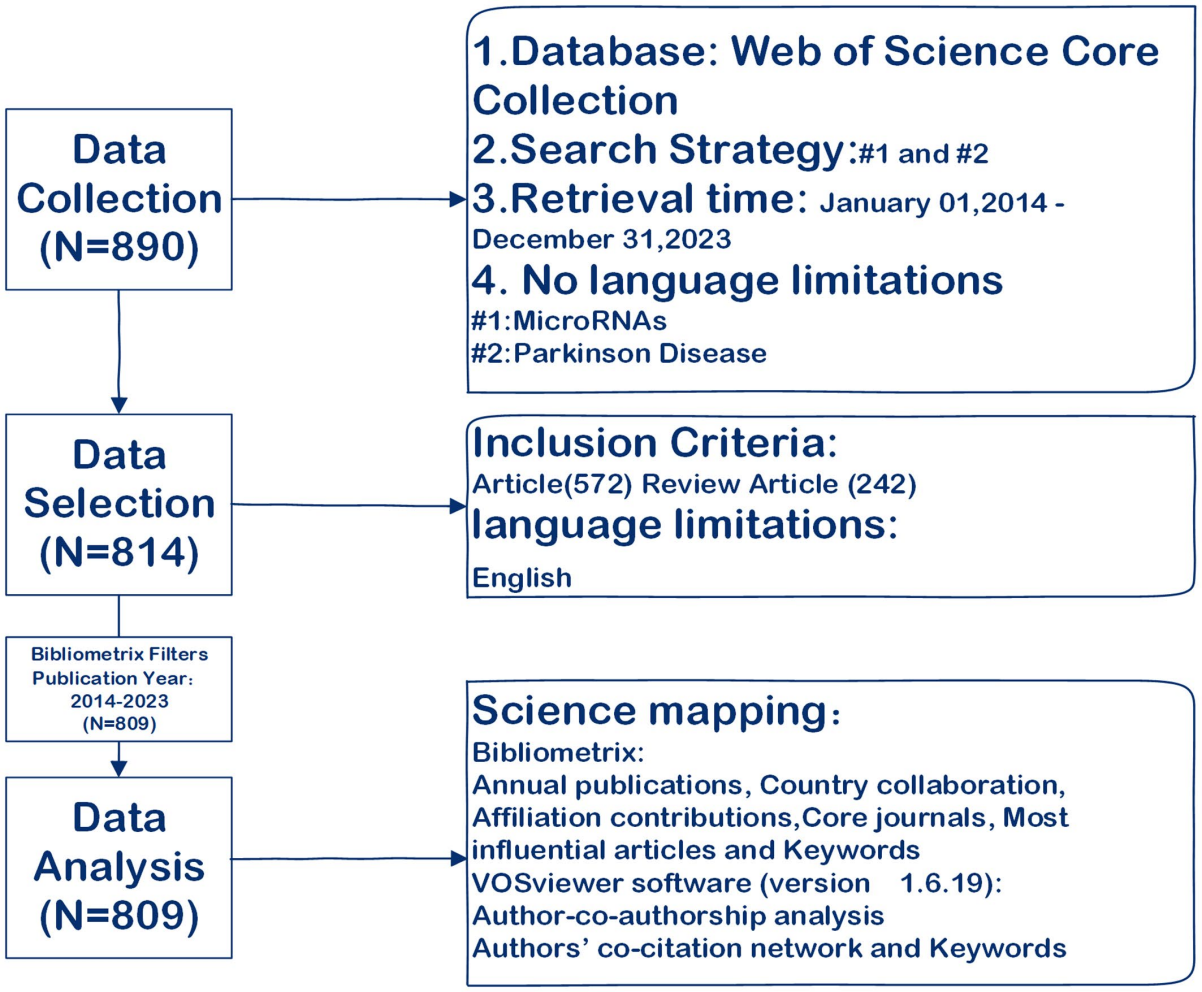
Flowchart of data collection and study design.

To provide a comprehensive overview of the thematic trends and development of the role of miRNAs in Parkinson’s disease, this study employed a systematic science mapping analysis based on the approach developed by Aria and Cuccurullo (19). To achieve this goal, we followed a standardized process for conducting science mapping analysis (19), which involved five key steps: research design, data collection, data analysis, data visualization, and interpretation. The analysis encompassed annual publications, country collaboration, affiliation contributions, authors, core journals, most influential articles, and keywords, utilizing the Bibliometrix R package (RStudio) (19). Authors’ co-citation networks and keywords were analyzed using VOSviewer software (version 1.6.19) (16). The study workflow is depicted in Figure 1.

## Results

### Summary of bibliographic data

A summary of the key statistics from the final literature dataset analyzed is presented below. Table 1 outlines the dataset used in this study, consisting of selected papers retrieved from WoSCC using the search criteria specified in the methodology section. The dataset included articles published between 2014 and 2023. There were 809 documents, with an average of 31.59 citations and an average interval of 4.44 years between data publications. A total of 315 journals covered miRNAs and PD, demonstrating an annual growth rate of 12.67%. The dataset comprised 1,796 author keywords and 1,976 keywords Plus. A total of 4,160 authors were represented, with an average of 6.59 co-authors per publication. The proportion of international collaborations among authors was 20.64%.

**Table 1:** Main bibliographic information about the final dataset of miRNAs and Parkinson’s disease.

Description	Results	Description	Results
MAIN INFORMATION ABOUT DATA		AUTHORS	
Timespan	2014:2023	Authors	4,160
Sources (Journals, Books, etc.)	315	Authors of single-authored docs	12
Documents	809	AUTHORS COLLABORATION	
Annual growth rate %	12.67	Single-authored docs	15
Document average age	4.44	Co-authors per doc	6.59
Average citations per doc	31.59	International co-authorships %	20.64
References	42,709		
DOCUMENT CONTENTS			
Keywords plus (ID)	1,976		
Author’s keywords (DE)	1,796		

### Trends in the number of published articles

**Figure 2:**
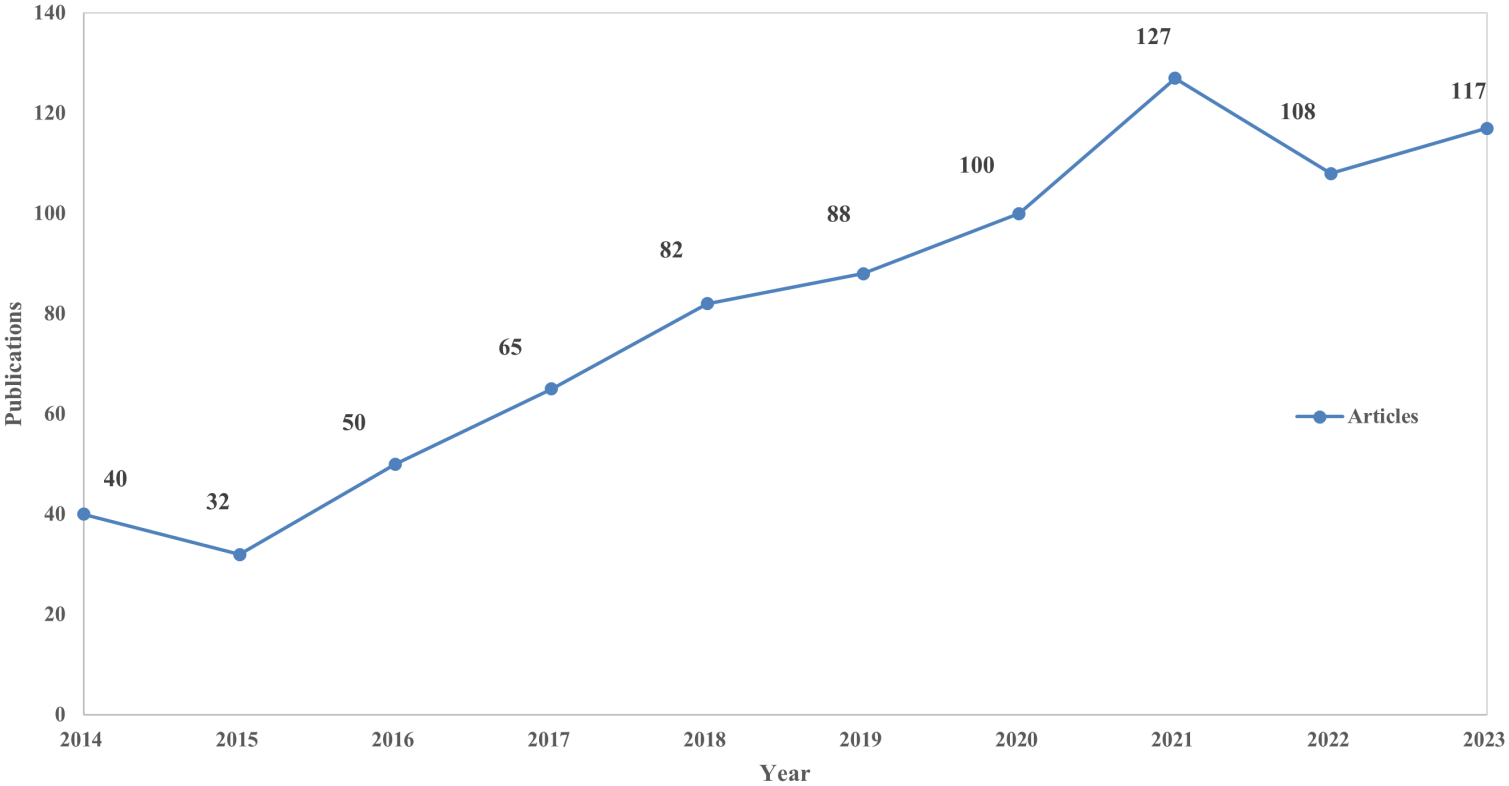
The annual number of publications and trends on miRNAs and Parkinson’s disease between 2014 and 2023.

The WoSCC database was queried for literature pertaining to non-motor symptoms in miRNAs and Parkinson’s disease from 2014 to 2023. The article type was restricted to “article” and “review article,” written in English, resulting in a total of 809 publications that met the inclusion criteria and were retrieved. Over the past decade, there has been a steady increase in articles discussing the non-motor symptoms of miRNAs and PD, indicating an overall upward trend. The trends illustrated in the number of published articles (Figure 2) suggest that ongoing research on miRNAs and Parkinson’s disease is increasingly attracting the attention of the global scientific community and has the potential to become a significant focus of future research endeavors.

### Countries and regions analysis

**Figure 3:**
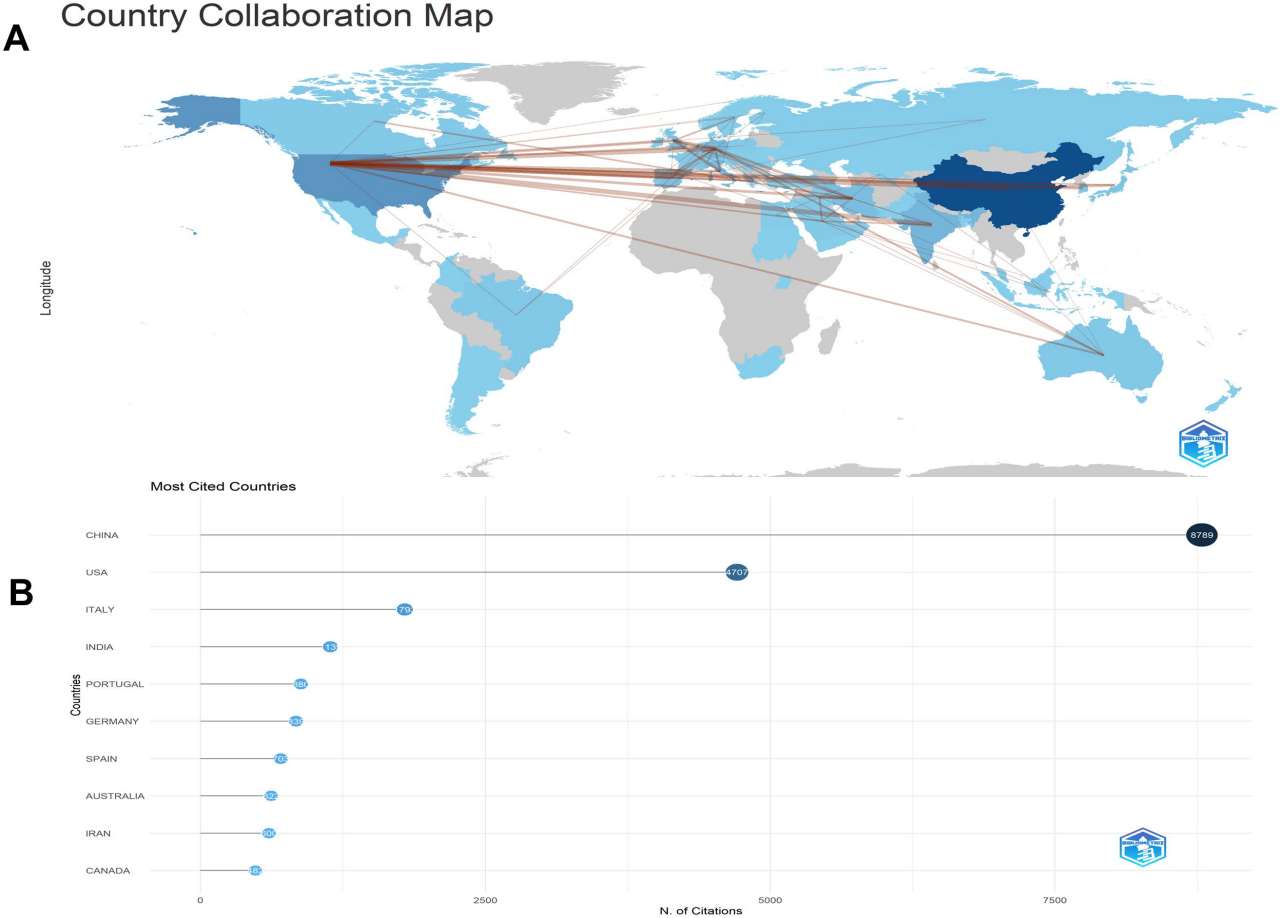
The cooperation of countries/regions contributed to publications on miRNAs and Parkinson’s disease between 2014 and 2023. **(A)** Country collaboration map. **(B)** Most cited countries. Analysis conducted using the Bibliometrix R package.

Our investigation identified research on microRNAs and Parkinson’s disease originating from 68 countries and regions worldwide. The collaborations between countries are illustrated in Figures 3A,B, and the top 10 countries are listed in Table 2. China led with the highest number of published studies at 344, followed by the United States (151), India (63), and Germany (54). Certain countries, including China, the United States, India, Germany, and Italy, exhibited high centrality in Figure 3A, indicating their significant involvement and contributions to research on this topic.

**Table 2:** Top 10 countries/regions and relevant institutions within the field of miRNAs and Parkinson’s disease.

Rank	Countries/regions	Count	Total citing articles	Average citations per item	H-index
1	China	344	4,978	24.75	47
2	USA	151	5,529	44.88	47
3	India	63	1,300	22.63	23
4	Germany	54	1,427	30.51	23
5	Italy	49	1,778	42.24	27
6	Iran	41	601	16.05	17
7	Spain	31	1,001	36.71	18
8	England	28	1,019	39.79	16
9	Japan	25	439	18.72	13
10	South Korea	19	554	29.58	9

### Affiliation analysis

**Figure 4:**
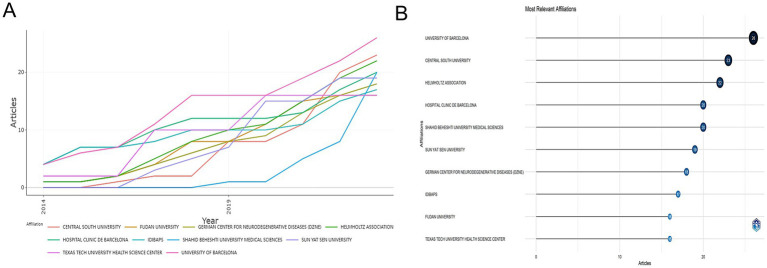
The cooperation of affiliation contributed to publications on miRNAs and Parkinson’s disease between 2014 and 2023. **(A)** Affiliations’ production over time. **(B)** Most relevant affiliation. Analysis conducted using the Bibliometrix R package.

Figure 4 illustrates the contributions of various affiliations to research on miRNAs and PD. The panels Figures 4A,B depict the temporal evolution of affiliation output and the most significant affiliation. The UNIVERSITY OF BARCELONA was ranked first, demonstrating a rapid increase in annual publications from four papers in 2014 to 26 articles in 2023. The HOSPITAL CENTRAL SOUTH UNIVERSITY and the HELMHOLTZ ASSOCIATION were ranked second and third, respectively. Although SHAHID BEHESHTI UNIVERSITY MEDICAL SCIENCES published their first relevant articles in 2019, they have shown a rapid growth in the number. The top 10 institutions in terms of relevant article publications since 2014 have consistently increased their research productivity.

### Analysis of influential authors

**Figure 5:**
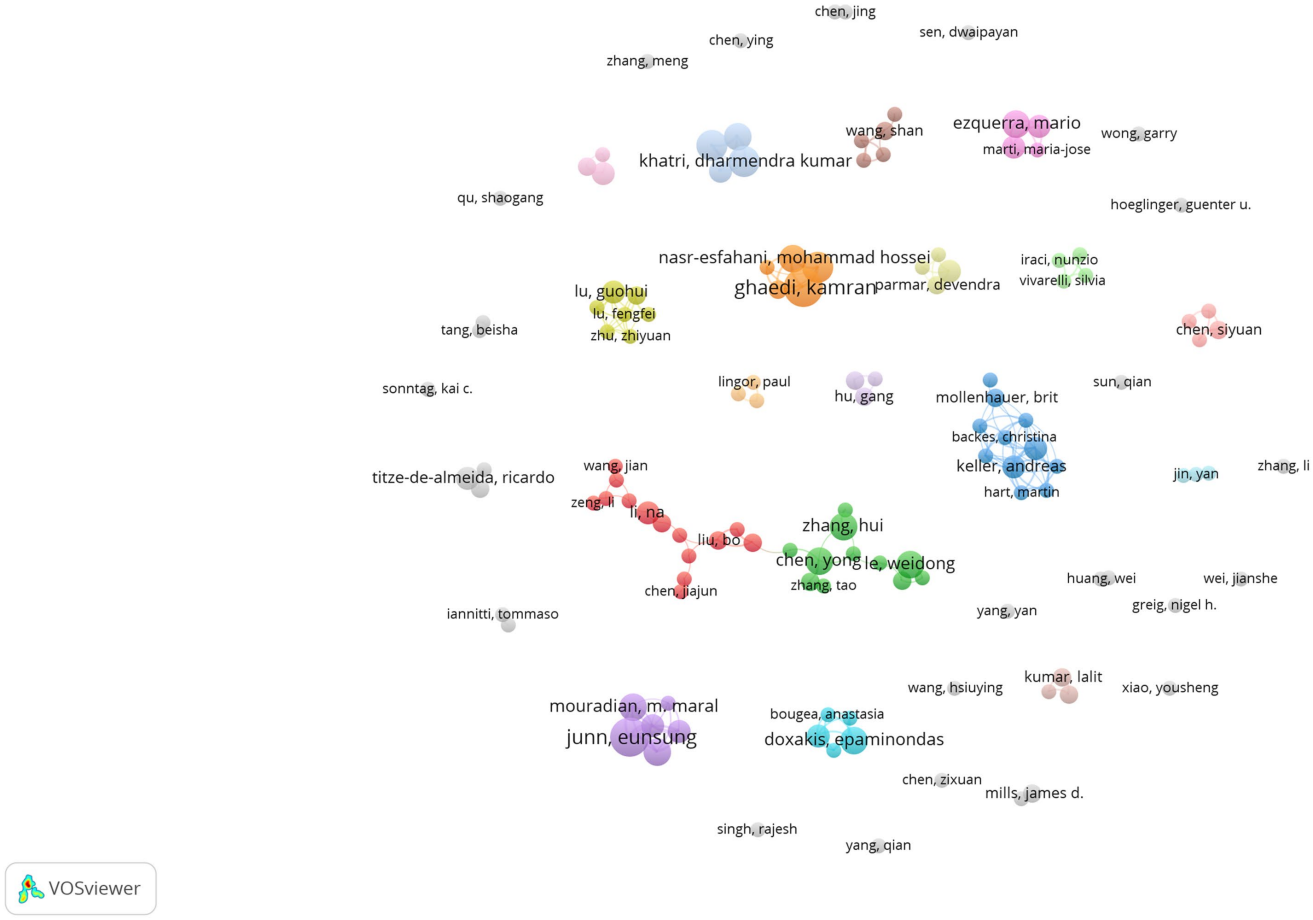
Author co-authorship analysis publications on miRNAs and Parkinson’s disease between 2014 and 2023. Analysis conducted using VOSviewer.

**Figure 6:**
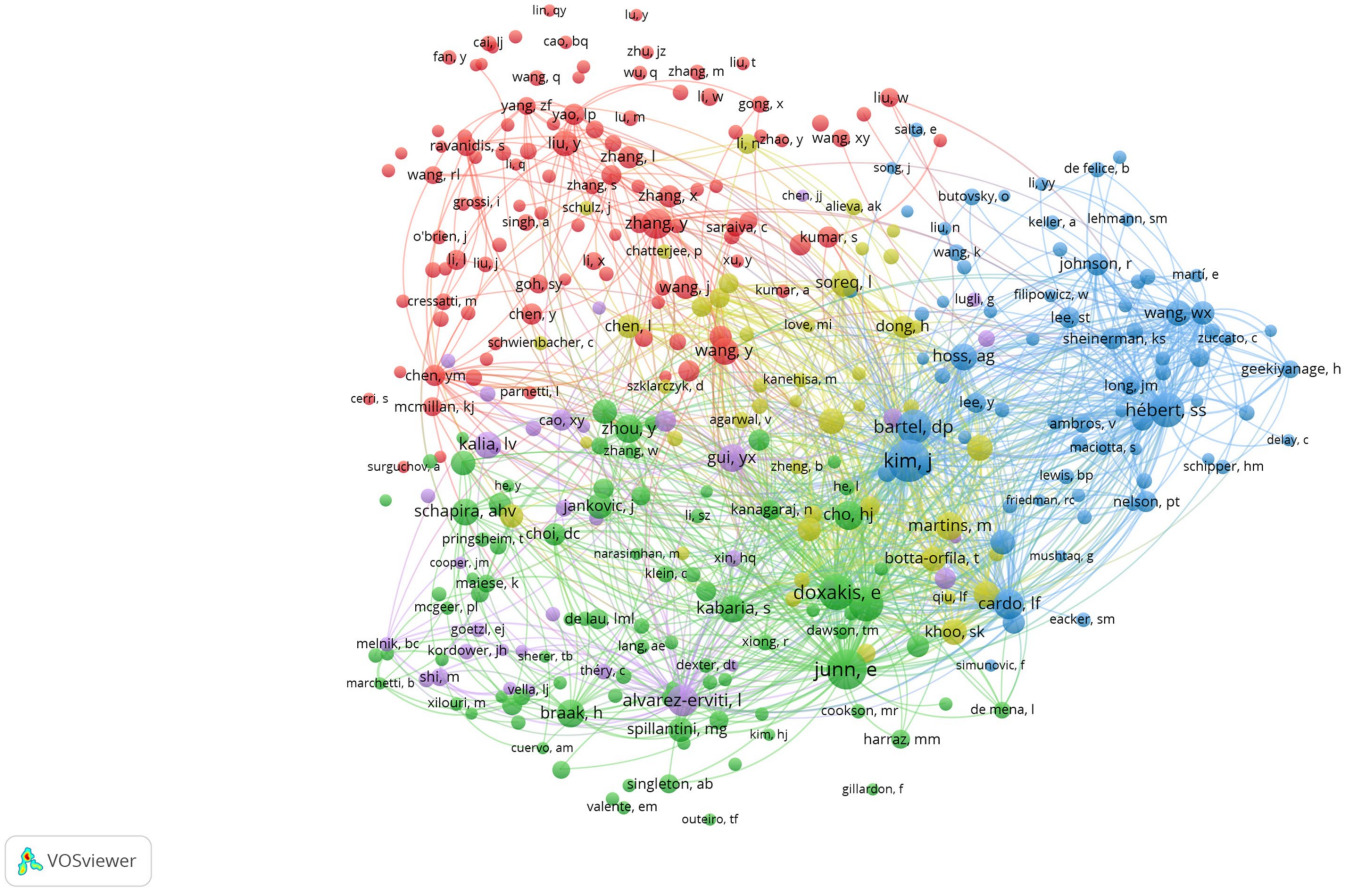
Authors’ co-citation network publications on miRNAs and Parkinson’s disease between 2014 and 2023. Analysis conducted using VOSviewer.

Figure 5 presents a visual representation of author co-authorship analysis. This network visualization reveals the distribution of collaborations and author groups through color and location. It facilitates an understanding of the network structure of academic collaborations and major research clusters. This analysis provides insights into collaboration patterns and helps identify high-impact authors within the field. High-impact collaboration groups, particularly the red and green groups, exhibit a higher level of collaboration and may represent pivotal research groups within the field. The interconnection between nodes of varying colors demonstrates the presence of interdisciplinary collaboration. Conversely, isolated nodes, such as those representing authors “*Sonntag, Kai C*” and “*Qu, Shaogang*,” suggest a lower likelihood of collaboration or indicate emerging researchers in the field. Co-citation analysis of authors focuses on the phenomenon of two authors being commonly cited by other articles (17). This analysis helps identify the knowledge structure, different research fields within a specific area, and the most influential or contributing researchers along with their interrelationships (20, 21). Figure 6 depicts the author co-citation network with five clusters. This network was generated using VOSviewer. The analysis included only authors with a minimum of 20 citations, resulting in 348 authors out of the total 30,359. Authors appearing in the same cluster are more likely to be cited together in published papers. The red cluster displays the highest number of authors, including *Zhang, Y, Chen, YM, Wang, Y, Yao, LP,* and *Liu, Y.* Conversely, the purple cluster has the fewest authors, including *Alvarez-Erviti, L, Gui, YX, Cao, XY, Kalia, LV, and Valadi, H. Notably*, the most frequently co-cited authors in the field include *Kim, J., Junn, E., Hébert, S.S.,* and *Doxakis, E*.

### Core journal analysis

**Figure 7:**
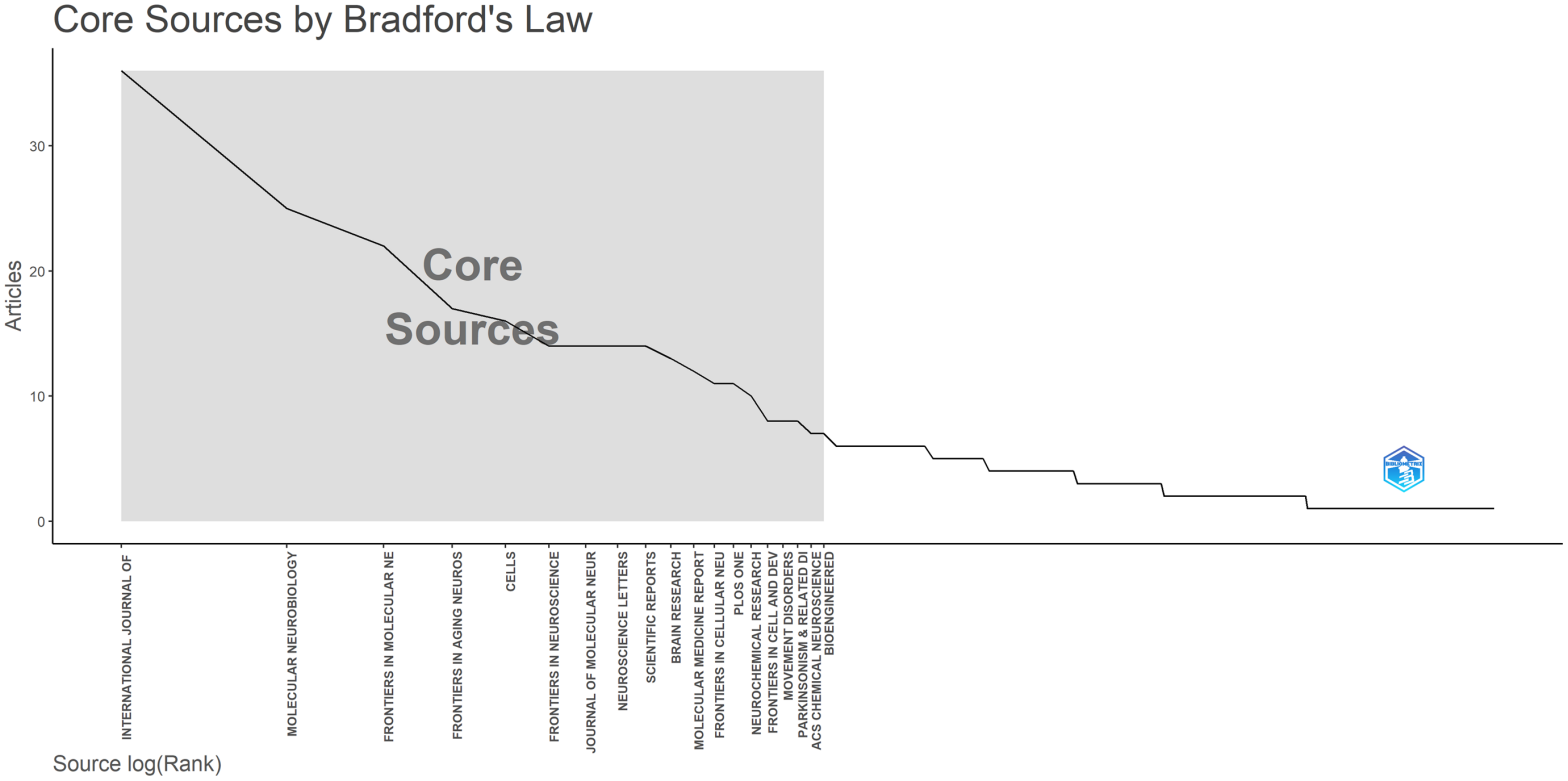
Distribution of core journals according to the principles of Bradford’s law. Analysis conducted using the Bibliometrix R package.

The contribution of journals to research in the field of miRNAs and Parkinson’s disease was next analyzed. Between 2014 and 2023, 809 publications were published in 315 journals. Table 3 lists the top 10 most prolific journals along with their overall citation counts, H-index, and earliest published year. The journals listed in Table 3 and Figure 7 contribute 185 articles, amounting to 22.9% of the dataset’s total of 809 publications. The INTERNATIONAL JOURNAL OF MOLECULAR SCIENCES (Articles = 36) topped the list, representing approximately 4% of all publications, followed closely by MOLECULAR NEUROBIOLOGY (Articles = 25), FRONTIERS IN MOLECULAR NEUROSCIENCE (Articles = 22), and FRONTIERS IN AGING NEUROSCIENCE (Articles = 17). Other notable journals included CELLS, FRONTIERS IN NEUROSCIENCE, JOURNAL OF MOLECULAR NEUROSCIENCE, NEUROSCIENCE LETTERS, SCIENTIFIC REPORTS, and BRAIN RESEARCH, with 16, 14, 14, 14, 14, and 13 published articles, respectively. The INTERNATIONAL JOURNAL OF MOLECULAR SCIENCES was ranked first in the H-index and total citations (H-index = 18; TC = 1,150). The INTERNATIONAL JOURNAL OF MOLECULAR SCIENCES, JOURNAL OF MOLECULAR NEUROSCIENCE, and BRAIN RESEARCH were earlier publications (PY Start = 2014).

**Table 3:** Top 10 most productive publication sources within the field of miRNAs and Parkinson’s disease (ranking in the table is based on this refers to the number of papers included in a specific journal).

Source journal	Rank	Articles	h_index	Total citations	PY_start
International Journal of Molecular Sciences	1	36	18	1,150	2014
Molecular Neurobiology	2	25	12	879	2015
Frontiers in Molecular Neuroscience	3	22	14	601	2016
Frontiers in Aging Neuroscience	4	17	13	576	2016
Cells	5	16	10	321	2018
Frontiers in Neuroscience	6	14	8	548	2016
Journal of Molecular Neuroscience	7	14	9	271	2014
Neuroscience Letters	8	14	12	465	2016
Scientific Reports	9	14	9	254	2015
Brain Research	10	13	11	456	2014

### Analysis of most influential articles

Table 4 presents the top 10 most globally cited documents in the field of miRNAs and Parkinson’s disease. The papers are evaluated based on both local and global citations, with local citations reflecting citations within a specific dataset and global citations representing the total number of citations across all documents in a bibliographic database (22). Among the top papers, “Altered microRNA profiles in cerebrospinal fluid exosome in Parkinson disease and Alzheimer disease” (23) stood out with the highest number of both local (*n* = 95) and global (*n* = 379) citations, indicating its significant impact in the field. It presented a balanced LC/GC ratio (25.07%) and a high normalized local citation rate (5.89), suggesting its importance both locally and globally. Next, “Profiles of Extracellular miRNA in Cerebrospinal Fluid and Serum from Patients with Alzheimer’s and Parkinson’s diseases Correlate with Disease Status and Features of Pathology” (24) stood out with its relatively high 79 local citations, this paper, with LC/GC ratio (28.94%) and a normalized local citation ratio of 4.44, indicated its importance in the local research discussion. Besides, “microRNA-155 Regulates Alpha-Synuclein-Induced Inflammatory Responses in Models of Parkinson Disease” (25) maintained a high LC/GC ratio (39.13%) and normalized local citations (4.31), emphasizing its local impact. Despite having fewer local citations (72) compared to the top two, its relatively high ratios suggest that a large proportion of its global citations come from the local community. “Identification of blood serum micro-RNAs associated with idiopathic and LRRK2 Parkinson’s disease” (26) boasted the highest LC/GC ratio (68.18%) among the top 10 papers, highlighting its substantial local relevance despite lower global citations. This paper, with a global citation count of 110 and a normalized local citation rate of 4.21, indicated its strong impact and relevance in the local research community compared to its global impact. Finally, “microRNAs in Parkinson’s disease: From Pathogenesis to Novel Diagnostic and Therapeutic Approaches” (27) balanced global citations (157) with a strong local impact, as evidenced by its LC/GC ratio (41.40%) and normalized local citations (5.60). These papers collectively offer valuable insights into the role of microRNAs in Parkinson’s disease, spanning diagnostic biomarkers, pathogenesis, and therapeutic approaches. Their strong local impact, as reflected in the citation metrics, underscores their significance within the field of PD research.

**Table 4:** The top 10 most local cited documents within the field of miRNAs and Parkinson’s disease.

Title	Year	First author	Journal	Local citations	Global citations	LC/GC ratio (%)	Normalized local citations	Normalized global citations
Altered microRNA profiles in cerebrospinal fluid exosome in Parkinson disease and Alzheimer disease	2015	YaXing Gui	Oncotarget	95	379	25.07	5.89	3.80
Profiles of extracellular miRNA in cerebrospinal fluid and serum from patients with Alzheimer’s and Parkinson’s diseases correlate with disease status and features of pathology	2014	Kasandra Burgos	Kasandra Burgos	79	273	28.94	4.44	3.90
Identification of blood serum micro-RNAs associated with idiopathic and LRRK2 Parkinson’s disease	2014	Teresa Botta-Orfila	Journal of Neuroscience Research	75	110	68.18	4.21	1.57
microRNA-155 regulates alpha-synuclein-induced inflammatory responses in models of Parkinson disease	2016	Aaron D. Thome	The Journal of Neuroscience	72	184	39.13	4.31	3.12
Inhibition of miR-34b and miR-34c enhances α-synuclein expression in Parkinson’s disease	2015	Savan Kabaria	FEBS Letters	68	125	54.40	4.22	1.25
Identification of a panel of five serum miRNAs as a biomarker for Parkinson's disease	2016	Haixia Ding	Parkinsonism & Related Disorders	68	125	54.40	4.07	2.12
Identification of circulating microRNAs for the differential diagnosis of Parkinson’s disease and multiple system atrophy	2014	Annamaria Vallelunga	Frontiers in Cellular Neuroscience	66	140	47.14	3.71	2.00
microRNAs in Parkinson’s disease: from pathogenesis to novel diagnostic and therapeutic approaches	2017	Loredana Leggio	International Journal of Molecular Sciences	65	157	41.40	5.60	3.18
microRNA profiles in Parkinson’s disease prefrontal cortex	2016	Andrew G. Hoss	Frontiers in Aging Neuroscience	64	114	56.14	3.83	1.93
MiR-124 regulates apoptosis and autophagy process in MPTP model of Parkinson’s disease by targeting to Bim	2016	Huiqing Wang	Brain Pathology	61	146	41.78	3.65	2.47

### Keywords analysis

**Figure 8:**
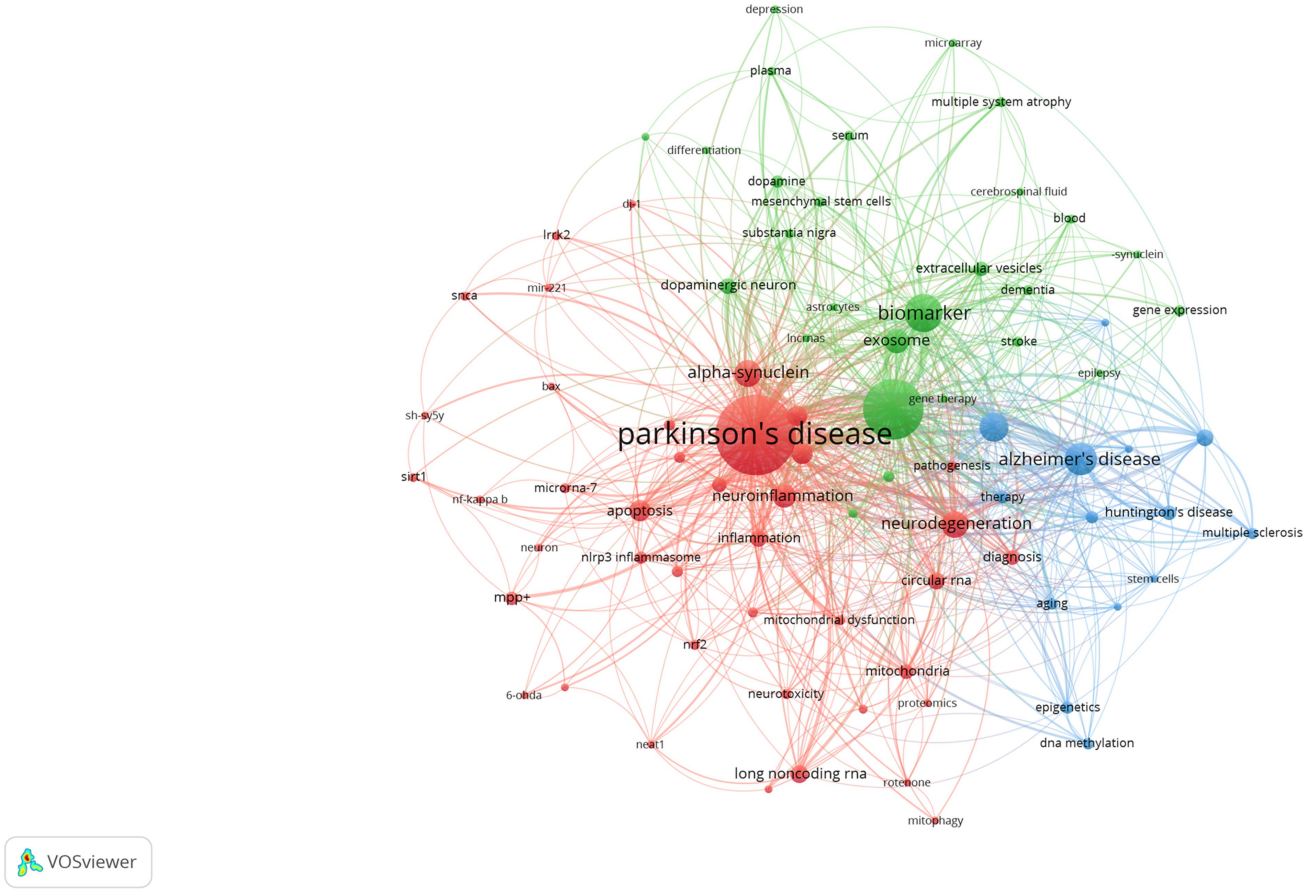
A network visualization map of co-occurring keywords using VOSviewer. Keywords in different fields are represented by dots of various colors. The size each dot indicates the frequency of occurrence. Analysis conducted using VOSviewer.

**Figure 9:**
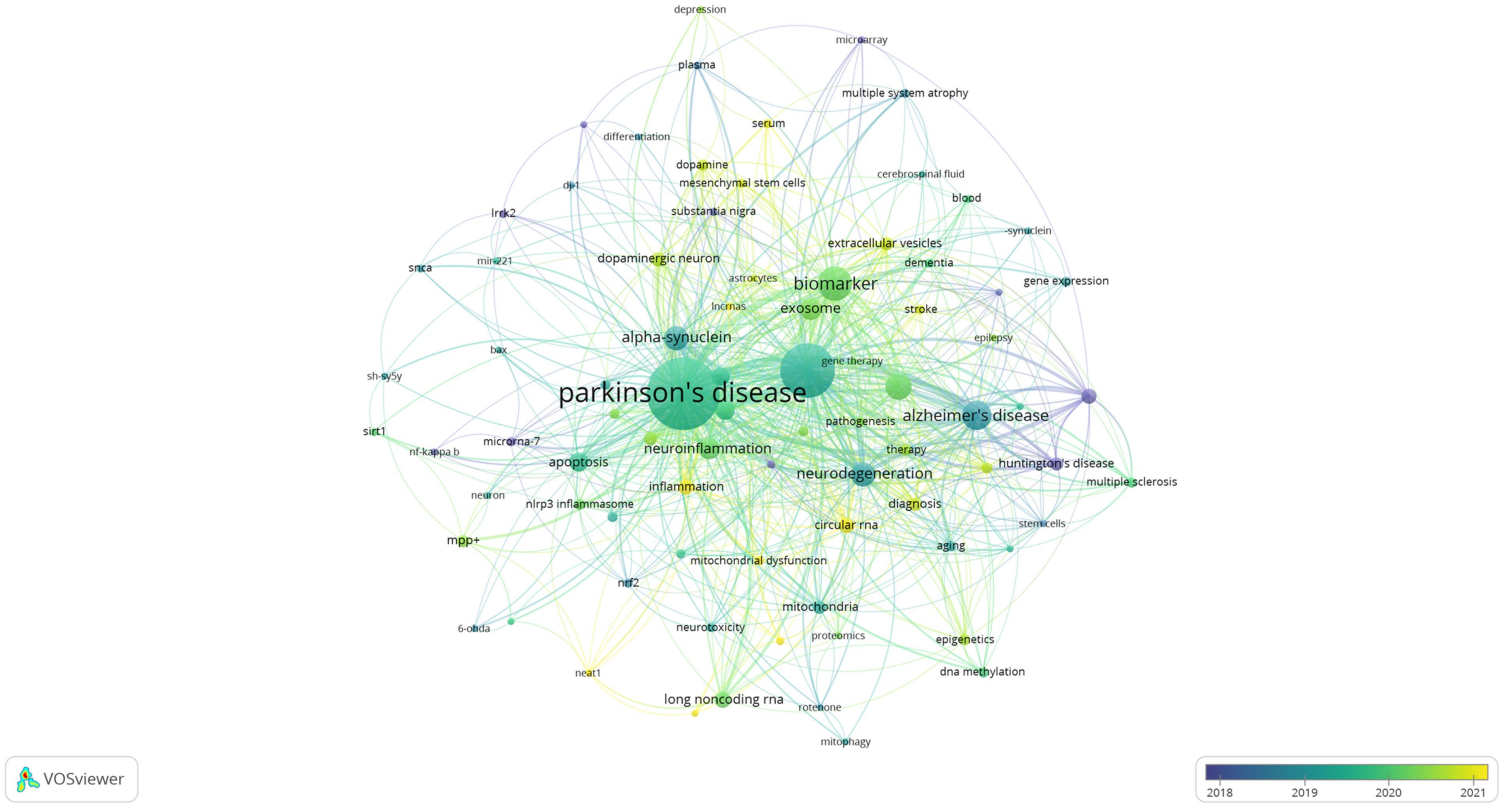
Analysis of research hot trend chart on the miRNAs and Parkinson’s disease, including an overlay visualization map of keywords using VOSviewer. The color gradient from blue to yellow indicates their chronological order of occurrence. Keywords closer to yellow represent more recent research trends, while those closer to blue represent earlier trends. Analysis conducted by VOSviewer.

**Figure 10:**
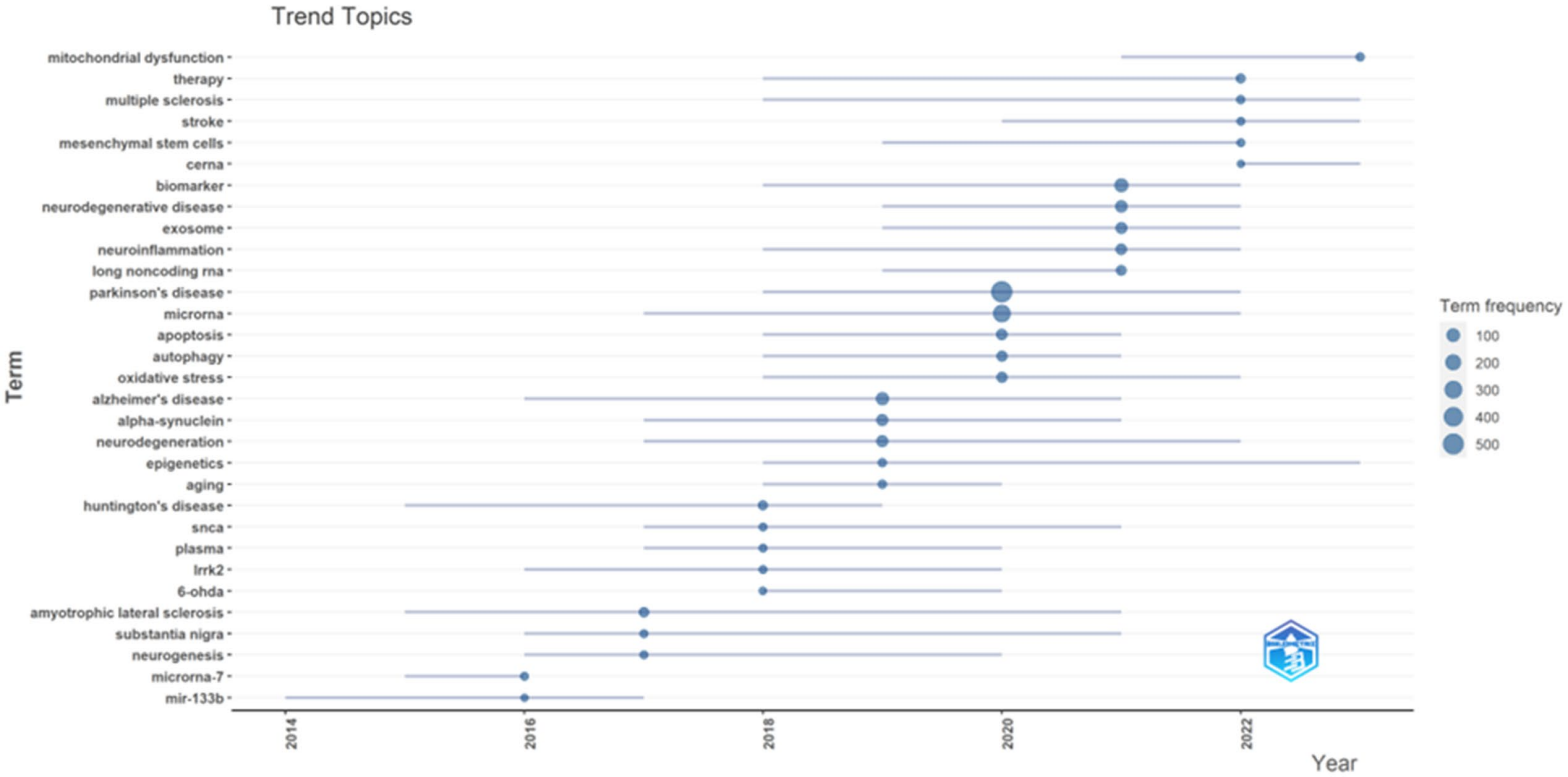
Trend topics based on words related to miRNAs and Parkinson’s disease research over time from 2014 to 2023, Analysis conducted by Bibliometrix R package.

The usage of “Author keywords” in the WoSCC database has increased substantially, suggesting its growing popularity within this field. Given the potential variability in results across different bibliometric tools, we employed a combined approach, utilizing VOSviewer’s keyword clustering map and Bibilometrix’s trend topics analysis to conduct an overlap analysis and identify research frontiers. In previous studies, keyword co-occurrence has been measured by the frequency with which two or more words appear together in the same document (28). This metric can be a valuable tool for identifying publication trends and knowledge evolution within a specific field (29). Following the consolidation of similar keywords, only those appearing five or more times within the WOSCC database were included in the final analysis. This process yielded a total of 82 keywords from an initial pool of 1,775 (Clustering set with Resolution: 1 and min. cluster size: 14). The co-occurrence network depicted in Figure 8 illustrates the clustering of these keywords into three primary groups. The Red node group centers on the molecular mechanisms and models of neurodegenerative diseases, with emphasis on PD. This group investigated key PD-associated proteins, including alpha-synuclein, DJ-1, LRRK2, and SNCA. Additionally, it explored neurotoxic substances like 6-OHDA and rotenone, commonly used to induce PD in animal models. The group also delved into various biological processes relevant to disease progression, such as neuroprotection, neurotoxicity, apoptosis, and mitochondrial dysfunction. The prominence of this group within the field underscored the substantial interest in this research area. The Green node group prioritized the study of specific cell types and cellular mechanisms, including astrocytes, microarray, and dopaminergic neurons. It investigated the role of cellular autophagy, neurotrophic factors, and neuroinflammation in neurodegenerative diseases. Furthermore, it utilized biological samples like plasma, serum, and cerebrospinal fluid for disease diagnosis and biomarker studies. The group’s research focused on a detailed examination of cell type-specific roles and biomarkers, as well as their potential implications for disease progression and diagnosis. The Blue node group highlighted emerging research areas like epigenetics, non-coding RNA, stem cells, and neurodegenerative diseases. These areas have attracted significant attention due to their potential for novel perspectives and therapeutic approaches. The emphasis on Therapy within the research indicates a strong commitment to developing effective treatments. Figure 9 presents an overlay visualization map of keywords generated using VOSviewer. The color spectrum from blue to yellow signifies the chronological order of keyword occurrence. Keywords depicted in colors closer to yellow represent more recent research associated with that particular term. Prominent keywords, visualized as larger circles, reflect the current direction of research and active hot topics within the field of miRNA research in Parkinson’s disease. These visualizations illustrate the progression of research in this area. Earlier studies focused on various neurodegenerative diseases, including Parkinson’s disease, Alzheimer’s disease, and amyotrophic lateral sclerosis (ALS). These investigations paid particular attention to the pathogenesis (disease development) of these conditions. Additionally, early research explored the role of alpha-synuclein in the pathogenesis of PD alongside mechanisms of inflammation and apoptosis. More recent studies have demonstrated a growing interest in the role of miRNAs in neurodegenerative diseases. Furthermore, other non-coding RNAs, such as circular RNAs and long non-coding RNAs, are now being incorporated into research efforts focused on their interactions with miRNAs. Current research delves into the potential of miRNAs as biomarkers for disease diagnosis, with particular emphasis on their role in neurodegenerative diseases. Additionally, studying dopaminergic neurons and oxidative stress remains a prominent area of interest, with researchers now incorporating modern molecular biology techniques and newly discovered mechanisms into their investigations. To identify trend topics within the study of miRNAs and Parkinson’s disease, keywords with a minimum occurrence of five were considered. For each year within the study period (2014–2023), the top five most frequent keywords are presented in Figure 10. The highlighted time range for each keyword in this figure indicates the period during which the keyword appeared at least five times among the keywords of articles published within a specific year. Additionally, the blue circles represent the overall frequency of the presented keywords from 2014 to 2023. These circles are positioned in alignment with the year in which the respective keywords appeared as one of the top five most frequent keywords. By observing the trend formed by these circles, it is possible to discern the evolution of the primary topics within the research domain of miRNAs and Parkinson’s disease. In this context, while the main trend topics in 2023 were “mitochondrial dysfunction,” the trend topics in 2022 included “therapy,” “multiple sclerosis,” “stroke,” “mesenchymal stem cells,” and “ceRNA.”

## Discussion

Over the past decade, miRNAs have gained significant momentum within the field of neurodegenerative diseases, particularly Parkinson’s disease (24, 27, 30). Our bibliometric analysis of 809 publications from the WoSCC database provides a comprehensive overview of research hotspots in this area. The substantial increase in publications since 2014 reflects the growing significance of this field, likely driven by an aging population, increased prevalence of PD, and advancements in scientific research techniques (29, 31). China has emerged as a leading force in this research domain, with institutions such as Central South University, Fudan University, and Sun Yat-sen University contributing significantly to the growing body of literature. This prominence can be attributed to increased government investment in scientific research, a large pool of talented researchers, and remarkable progress in biotechnology and genomics (32–34). The global research landscape, as visualized through our analysis, underscores the importance of data availability and accessibility in facilitating scientific progress. As the relationship between miRNAs and PD continues to be extensively investigated (35–37), our findings highlight the dynamic nature of this field and its potential for future breakthroughs in understanding and treating Parkinson’s disease.

A bibliometric analysis of core journals identified the top 10 journals, each publishing at least 13 papers with an H-index of 8 or higher, a metric that combines publication quantity and impact (38, 39). The cumulative number of citations serves as an indicator of research interest within specific domains. The International Journal of Molecular Sciences, with an H-index of 18 and 1,150 citations, emerged as the most frequently cited journal. This open-access, peer-reviewed publication, dedicated to molecular biology, biochemistry, and biophysics, fosters academic exchange through the dissemination of original research and review articles. Of particular note, the article titled “MicroRNAs in Parkinson’s disease: From Pathogenesis to Novel Diagnostic and Therapeutic Approaches” has significantly advanced the field by elucidating the roles of microRNAs as diagnostic biomarkers and therapeutic targets (27). Other influential studies included investigations into altered microRNA profiles in cerebrospinal fluid exosomes of patients with Parkinson’s and Alzheimer’s diseases, the regulation of alpha-synuclein-induced inflammatory responses by microRNA-155, and the identification of serum microRNAs linked to idiopathic and LRRK2-associated Parkinson’s disease (23–26). These contributions significantly advance our understanding of the molecular mechanisms underlying Parkinson’s disease, particularly the intricate relationship between miRNA dysregulation and disease pathology. Moreover, they provide valuable insights for future research and clinical applications.

Keywords serve as the core content of an article, and their frequencies offer valuable insights into the predominant trends within a research area (40). Beyond “Parkinson’s disease,” “microRNA” emerged as the most frequently used keyword in the field of miRNAs and Parkinson’s disease. Indeed, miRNAs play a crucial role in cell differentiation, development, the regulation of the cell cycle, and apoptosis. They are also implicated in the pathology of numerous diseases. It is now understood that miRNAs also regulate genes associated with PD (34). “Alpha-synuclein” is a frequently used keyword in PD research. A hallmark of PD is the accumulation of alpha-synuclein in the brain, accompanied by signs of inflammation and immune activation (41). Alpha-synuclein aggregation triggers oxidative stress and inflammation in neurons and induces apoptosis and autophagy dysregulation (42). Collectively, these pathological processes lead to neuronal dysfunction and death, ultimately resulting in the clinical symptoms of Parkinson’s disease. Certain miRNAs are considered key inflammation-initiating molecules and could potentially serve as targets for PD therapeutics (25, 37). The role of miRNAs in neurodegenerative diseases, such as PD, is garnering increasing attention (37). Studies have demonstrated the potential of miRNAs as biomarkers for early diagnosis and monitoring of disease progression (7). Additionally, circular RNA (circRNA) and long noncoding RNA (lncRNA) also play significant roles in the pathological mechanisms of PD (4, 43). These noncoding RNAs further influence neuronal survival and function by regulating miRNA function. The intricate interactions between these noncoding RNAs offer novel perspectives for understanding the molecular mechanisms of PD and are anticipated to provide innovative strategies for the diagnosis and treatment of the disease (4). In recent years, the role of exosomes and other extracellular vesicles (EVs) in neurodegenerative diseases has attracted considerable attention. These extracellular vesicles play a vital role in intercellular communication and may carry disease-related biomarkers, thus providing new avenues for early diagnosis and treatment of diseases (44). It has been established that in PD, exosomes can transport *α*-synuclein, facilitating its propagation in the brain and exacerbating neuronal damage and disease progression (31). Consequently, investigating the mechanisms of exosomes and extracellular vesicles in PD will contribute to a deeper understanding of the disease’s pathological process and the development of novel diagnostic and therapeutic approaches.

Another trend identified in the keyword analysis was “ceRNA” and “mitochondrial dysfunction.” ceRNAs are RNA molecules that regulate gene expression through competitive binding of miRNAs, including lncRNAs, circRNAs, and mRNAs (45). ceRNA networks play a crucial role in numerous biological processes and diseases, including neurodegenerative diseases such as Parkinson’s disease (46). In PD, the ceRNA network may influence disease onset and progression by modulating gene expression associated with mitochondrial function, oxidative stress, neuroinflammation, and synaptic function (46, 47). For instance, certain lncRNAs and circRNAs can increase the expression of target mRNAs by binding to specific miRNAs and decreasing the repression of these miRNAs on their target mRNAs. This regulatory mechanism can affect the expression and function of PD-related genes such as *SNCA* (encoding *α*-synuclein), *LRRK2*, and *PINK1* (46). In PD, mitochondrial dysfunction is recognized as one of the key mechanisms involved in disease pathogenesis and progression. Impaired energy metabolism, increased oxidative stress, and induction of apoptosis within mitochondria are all closely associated with neuronal damage in PD (48). Moreover, studies have demonstrated that mRNA expression and translational regulation in PD are also intimately linked to mitochondrial dysfunction. Abnormal expression of certain mRNAs may lead to abnormal synthesis of mitochondrial proteins, which can further exacerbate mitochondrial dysfunction. For example, mutations in the *PINK1* and *Parkin* genes affect mitochondrial quality control, leading to mitochondrial damage and neuronal death (4). By studying PD-associated miRNA expression profiles, we can gain valuable insights into the molecular mechanisms of the disease and develop novel diagnostic markers and therapeutic targets.

## Limitations

We acknowledge certain limitations within our research. Indeed, our data collection encompassed a broad temporal range (2014–2023), which could have resulted in missing data, potentially introducing publication bias that might have influenced the outcome of our analysis. Besides, to uphold a high standard for bibliometric evaluation, our study relied exclusively on entries within the WoSCC database, as a premier repository for scholarly articles across various research disciplines. Consequently, many studies were excluded due to their publication in non-SCI indexed journals or other databases. Moreover, the tools employed, such as the Bibliometrix R package and VOSviewer, may not be exhaustive in their retrieval capabilities. These limitations could exert a marginal influence on the study’s findings but are not anticipated to significantly alter the primary patterns reported in this manuscript. In summary, our investigation provides a foundation for understanding the research themes, focal points, and evolutionary trajectories within the domain of miRNAs and Parkinson’s disease.

## Conclusion

In summary, miRNA research in PD pathogenesis, diagnostic markers, and therapeutic strategies remains the primary hotspot within this field. However, emerging research directions and technical approaches, including ceRNAs, mitochondrial function, and stem cells, are gradually gaining attention. Future research in this area may continue to delve into the mechanisms of miRNAs while simultaneously integrating emerging technologies and interdisciplinary research to generate novel ideas and strategies for the early diagnosis, treatment, and prognosis of Parkinson’s disease. The findings of this study can assist researchers in staying abreast of the latest developments in this field and determining the direction of future research within the domain.

## Data Availability

The original contributions presented in the study are included in the article/Supplementary material, further inquiries can be directed to the corresponding author.
