# Supplementary material for: Bibliometric analysis of microRNAs and Parkinson’s disease from 2014 to 2023
Source: Front Neurol. 2024 Sep 25;15:1466186. doi: 10.3389/fneur.2024.1466186 (PMC11462628; doi:10.3389/fneur.2024.1466186)
Supplement: Supplementary file 2 [file Table_1.DOCX]

**Supplement Document 1: Mesh Terms and Search Strategy**

Literature data were collected from the Science Citation Index Expanded (SCIE). For the sake of comprehensiveness, Mesh Terms identifies the search subject term. as search #1 AND #2

#1((TI=("MicroRNAs" OR "MicroRNA" OR "miRNAs" OR "Micro RNA" OR "RNA, Micro" OR "miRNA" OR "Primary MicroRNA" OR "MicroRNA, Primary" OR "Primary miRNA" OR "miRNA, Primary" OR "pri-miRNA" OR "pri miRNA" OR "RNA, Small Temporal" OR "Temporal RNA, Small" OR "stRNA" OR "Small Temporal RNA" OR "pre-miRNA" OR "pre miRNA")) OR AB=("MicroRNAs" OR "MicroRNA" OR "miRNAs" OR "Micro RNA" OR "RNA, Micro" OR "miRNA" OR "Primary MicroRNA" OR "MicroRNA, Primary" OR "Primary miRNA" OR "miRNA, Primary" OR "pri-miRNA" OR "pri miRNA" OR "RNA, Small Temporal" OR "Temporal RNA, Small" OR "stRNA" OR "Small Temporal RNA" OR "pre-miRNA" OR "pre miRNA")) OR AK=("MicroRNAs" OR "MicroRNA" OR "miRNAs" OR "Micro RNA" OR "RNA, Micro" OR "miRNA" OR "Primary MicroRNA" OR "MicroRNA, Primary" OR "Primary miRNA" OR "miRNA, Primary" OR "pri-miRNA" OR "pri miRNA" OR "RNA, Small Temporal" OR "Temporal RNA, Small" OR "stRNA" OR "Small Temporal RNA" OR "pre-miRNA" OR "pre miRNA")

#2((TI=("Parkinson Disease" OR "Idiopathic Parkinson's Disease" OR "Lewy Body Parkinson's Disease" OR "Parkinson's Disease, Idiopathic" OR "Parkinson's Disease, Lewy Body" OR "Parkinson Disease, Idiopathic" OR "Parkinson's Disease" OR "Idiopathic Parkinson Disease" OR "Lewy Body Parkinson Disease" OR "Primary Parkinsonism" OR "Parkinsonism, Primary" OR "Paralysis Agitans")) OR AB=("Parkinson Disease" OR "Idiopathic Parkinson's Disease" OR "Lewy Body Parkinson's Disease" OR "Parkinson's Disease, Idiopathic" OR "Parkinson's Disease, Lewy Body" OR "Parkinson Disease, Idiopathic" OR "Parkinson's Disease" OR "Idiopathic Parkinson Disease" OR "Lewy Body Parkinson Disease" OR "Primary Parkinsonism" OR "Parkinsonism, Primary" OR "Paralysis Agitans")) OR AK=("Parkinson Disease" OR "Idiopathic Parkinson's Disease" OR "Lewy Body Parkinson's Disease" OR "Parkinson's Disease, Idiopathic" OR "Parkinson's Disease, Lewy Body" OR "Parkinson Disease, Idiopathic" OR "Parkinson's Disease" OR "Idiopathic Parkinson Disease" OR "Lewy Body Parkinson Disease" OR "Primary Parkinsonism" OR "Parkinsonism, Primary" OR "Paralysis Agitans")
